# Diversity of *Campylobacter* spp. circulating in a rhesus macaque (*Macaca mulatta*) breeding colony using culture and molecular methods

**DOI:** 10.1128/msphere.00560-24

**Published:** 2024-10-23

**Authors:** Rebecca L. Bacon, Carolyn L. Hodo, Jing Wu, Shannara Welch, Colette Nickodem, Javier Vinasco, Deborah Threadgill, Stanton B. Gray, Keri N. Norman, Sara D. Lawhon

**Affiliations:** 1 Department of Veterinary Pathobiology, School of Veterinary Medicine & Biomedical Sciences, Texas A&M University, College Station, Texas, USA; 2 Michale E. Keeling Center for Comparative Medicine and Research, The University of Texas MD Anderson Cancer Center, Bastrop, Texas, USA; 3 Veterinary Medical Teaching Hospital, School of Veterinary Medicine & Biomedical Sciences, Texas A&M University, College Station, Texas, USA; 4 Department of Pathobiological Sciences, University of Wisconsin—Madison, Madison, Wisconsin, USA; 5 Department of Cell Biology and Genetics, School of Medicine, Texas A&M University, College Station, Texas, USA; 6 Department of Veterinary Integrative Biosciences, School of Veterinary Medicine & Biomedical Sciences, Texas A&M University, College Station, Texas, USA; University of Michigan—Ann Arbor, Ann Arbor, Michigan, USA

**Keywords:** *Campylobacter*, rhesus macaque, *Campylobacter jejuni*, *Campylobacter coli*, chronic enterocolitis, post-infectious irritable bowel, macaque, diarrhea

## Abstract

*Campylobacter jejuni* and *Campylobacter coli* represent the leading causes of bacterial gastroenteritis in humans, and infections can produce post-infectious irritable bowel syndrome (PI-IBS). Rhesus macaques (*Macaca mulatta*) (RM) are similarly susceptible to acute campylobacteriosis and represent a potential model of PI-IBS. We characterized the *Campylobacter* species circulating in an RM breeding colony using culture, qPCR, and whole genome sequencing (WGS). We also compared the *C. jejuni* and *C. coli* prevalence in RM as detected with qPCR versus culture and identified risk factors for bacteria presence and intestinal disease. Culture of 275 samples yielded *C. coli* (103) and *C. jejuni* (8), of which 21.6% were resistant to quinolones and 3.6% were resistant to macrolides. Multidrug-resistant isolates were obtained exclusively from animals exhibiting diarrhea or with histologically confirmed chronic enterocolitis. WGS revealed a non-clonal population of *Campylobacter* spp. Genotypic predictions of resistance were excellent except for aminoglycosides. All sequenced isolates contained genes for all subunits of cytolethal distending toxin. qPCR detected a prevalence of 45.9% for *C. coli* and 29.6% for *C. jejuni*. The quantity of either bacteria was significantly higher (*P* < 0.05) in animals with intestinal disease compared to healthy animals, though only young age was significantly associated with the presence of *Campylobacter* sp. or intestinal disease. Significantly more *C. jejuni* positive animals were detected with qPCR than with culture. These results provide a comprehensive characterization of *Campylobacter* spp. circulating in a breeding colony of RM in the United States and suggest that qPCR is superior for the detection of *C. jejuni* in RM.

**IMPORTANCE:**

Gastrointestinal disease is one of the most common reasons for hospitalization in non-human primate colonies and accounts for over one-third of non-research related euthanasia. In rhesus macaques, this manifests as both acute diarrhea and chronic enterocolitis (CE), a syndrome of chronic diarrhea resulting in poor weight gain or weight loss which is minimally responsive to treatment. *Campylobacter* spp. are major causes of acute enterocolitis in rhesus macaques and may predispose individuals to the development of CE, similar to post-infectious irritable bowel syndrome in humans. Despite these concerns, there are few studies characterizing *Campylobacter* in rhesus macaque colonies, in particular utilizing whole genome sequencing and assessing findings with respect to the health status of the host. Our findings provide insight into *Campylobacter* strains circulating in rhesus macaque colonies, which can improve clinical monitoring, assist in treatment decisions, and provide new avenues of investigation into campylobacteriosis as a catalyst for CE.

## INTRODUCTION

*Campylobacter* are Gram-negative, microaerophilic, spiral-shaped bacteria that can be commensal or pathogenic in the gastrointestinal tract of birds and mammals, with *C. jejuni* and *C. coli* representing the leading causes of bacterial gastroenteritis in humans worldwide (1–3). *Campylobacter jejuni* is a known pathogen of rhesus macaques (*Macaca mulatta*) but there is some debate over the pathogenicity of *C. coli* in this species (4–7). Prevalence estimates of *Campylobacter-*infected rhesus macaques range between 45% and 97% of individuals, with most identifiable isolates classified as *C. coli* and fewer as *C. jejuni* and *C. lari* (5, 8–10). Campylobacteriosis in rhesus macaques is classically associated with acute bacterial enterocolitis. However, *Campylobacter* infections in humans are notorious for resulting in post-infectious sequelae, including Guillain-Barré syndrome, reactive arthritis, and post-infectious irritable bowel syndrome (PI-IBS) (11–14). PI-IBS is a syndrome of chronic, low-grade intestinal inflammation resulting in abdominal pain and stool disturbances, with up to 20% of individuals developing PI-IBS following an episode of *Campylobacter*-associated enteritis (2, 3, 15). Numerous host factors play a role in whether an individual develops PI-IBS following infection, but some studies suggest *Campylobacter* strain-specific differences which are more likely to produce PI-IBS. Toxigenic strains in particular have been implicated, and the B subunit of cytolethal distending toxin (CdtB) has been proposed to be a catalyst for disease as a molecular mimic of the host protein vinculin (16–18). Whole genome sequencing (WGS) also demonstrated strains with specific variations in the expression of genes associated with bacterial stress response and core biosynthetic pathways to be more likely to result in PI-IBS (19).

Gastrointestinal disease in rhesus macaques is one of the most common reasons for hospitalization and accounts for up to 33% of non-research related euthanasias (20). In addition to acute enterocolitis, often *Campylobacter*-associated, rhesus macaques experience a syndrome termed chronic enterocolitis (CE), otherwise known as idiopathic chronic diarrhea. CE is a syndrome of chronic diarrhea resulting in poor weight gain and failure to thrive and often leads to euthanasia due to welfare concerns (4, 13, 21–24). There is growing suspicion that *Campylobacter* spp. infections may predispose rhesus macaques to the development of CE, similar to PI-IBS in humans (23, 25). Despite this, few studies have surveyed the characteristics of *Campylobacter* spp. circulating in rhesus macaque colonies, and even fewer have used WGS (5, 8, 10, 26, 27). Defining the role of *Campylobacter* spp. in the development of CE and characterizing the role of *C. coli* in both acute and chronic disease in rhesus macaques may facilitate the identification of controllable risk factors and therapeutic targets. This would result in the reduction of morbidity and mortality associated with both acute colitis and CE (5, 28). Detailed characterization could also support the utilization of CE in rhesus macaques as a natural model for PI-IBS in humans.

Diagnosis of campylobacteriosis in rhesus macaques is typically limited to culture methods. However, for many bacteria, molecular methods of detection such as quantitative PCR (qPCR) are superior to culture, particularly when animals are shedding low numbers of bacteria (29, 30). qPCR may also provide a rapid method for quantifying bacterial loads which could be associated with the disease status of the host. In this study, we validate the use of qPCR for *C. coli* and *C. jejuni* detection in rectal swabs from rhesus macaques. The qPCR assays, traditional culture methods, and WGS of obtained isolates were used to compare the *Campylobacter* spp. affecting healthy rhesus macaques and those with acute colitis and CE. These findings will provide clinicians with a rapid, reliable diagnostic technique for *C. jejuni* and *C. coli* in rhesus macaques and lend insight into antimicrobial susceptibility, bacterial quantity, and strain differences which may play a role in the health status of the host animal.

## RESULTS

We collected 277 samples from 266 animals with five animals sampled at multiple time points. Of those, 240 samples had paired culture and PCR swabs, 35 samples had only a culture swab, and 2 had only a qPCR swab. Fifty-eight samples from 50 individuals were obtained from animals with intestinal disease at the time of sample collection. Animals with known intestinal disease included animals with acute colitis, CE, colonic carcinoma, and intestinal amyloidosis. The age, sex, and disease status of sampled animals are shown in Table 1.

**TABLE 1:** Population summary of sampled animals by age, sex, and health status at the time of first sampling

	Health status	Juvenile^*a*^	Peripubertal^*a*^	Young adult^*a*^	Adult^*a*^	Total
Male	Healthy	11	17	11	16	55
	Acute colitis	1	0	0	0	1
	CE	9	2	2	4	17
	Developed CE^*b*^	0	1	0	0	1
	Other	1	0	1	1	3
	Total male	22	20	14	21	77
Female	Healthy	11	35	69	43	158
	Acute colitis	4	2	0	1	7
	CE	6	3	0	6	15
	Developed CE^*b*^	0	3	0	1	4
	Other	0	0	0	5	5
	Total female	21	43	69	56	189
Total		43	63	83	77	266

^*a*^ Juvenile, ≤2 years of age; peripubertal, 3–4 years of age; young adult, 4–9 years of age; adult, ≥10 years of age.

^*b*^ Animals developed CE following date of sampling.

### Culture results

**Fig 1:**
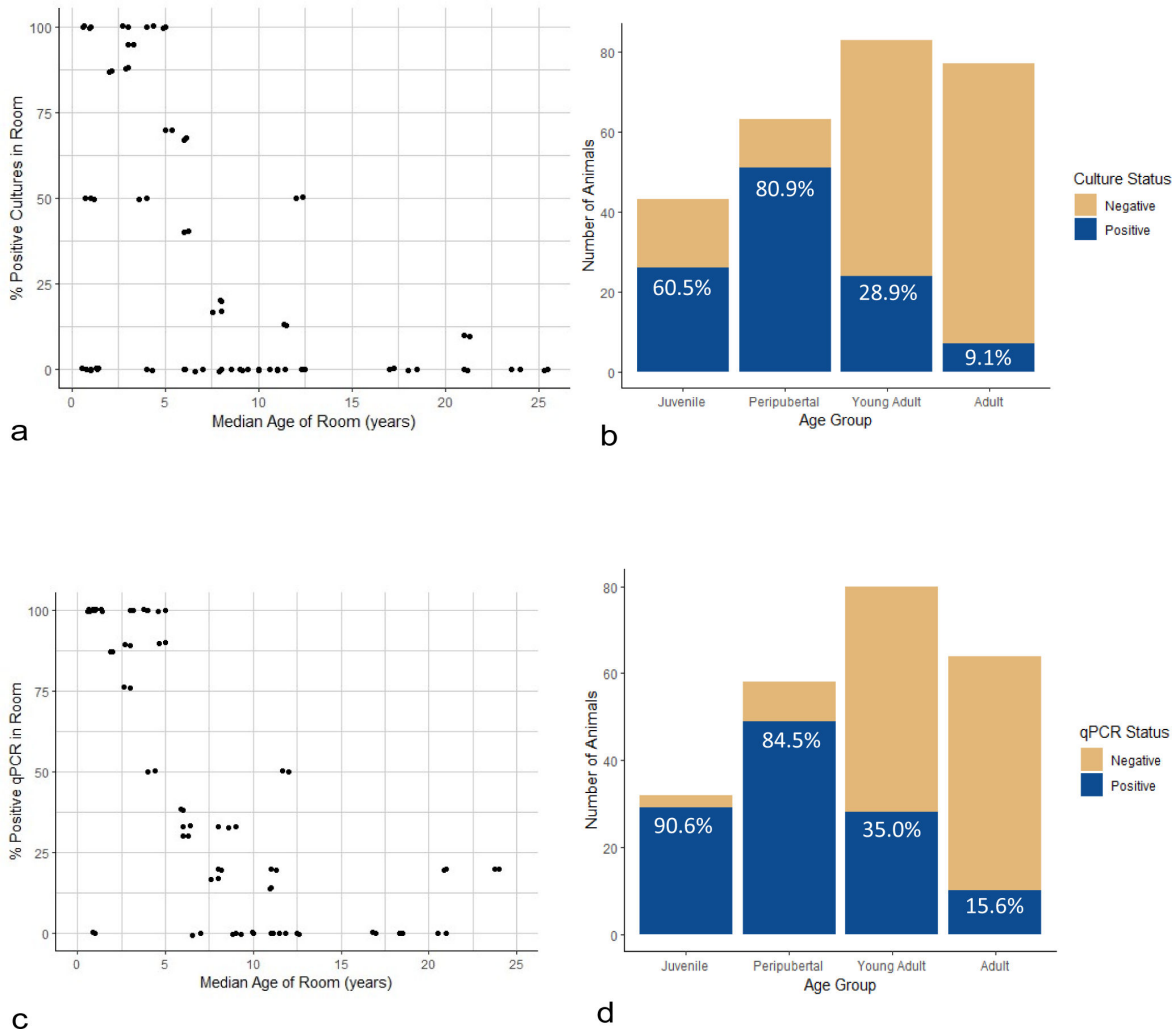
Distribution of *Campylobacter* spp. culture or qPCR positive animals. (**a**) Each data point represents a unique room location, plotted against the median age of the room and *Campylobacter* spp. prevalence using culture in the room. (**b**) Number of animals culture positive for either *C. jejuni* or *C. coli* separated by age group. (**c**) Each data point represents a unique room location, plotted against the median age of the room and *Campylobacter* spp. prevalence using qPCR in the room. (**d**) Number of animals qPCR positive for either *C. jejuni* or *C. coli* separated by age group. Juvenile animals are 2 or less years old, peripubertal animals are 3 or 4 years old, young adult animals are 5 years old, and adult animals are 10 or more years old.

Culture of 275 rectal swabs yielded 115 *Campylobacter* sp. isolates (41.8% prevalence). However, three isolates were not frozen for further analysis and one isolate could not be recovered following freezing, resulting in 111 *Campylobacter* sp. isolates available for further study and the reduction of the overall sample size to 271 for further prevalence calculations. Eighty-six isolates were from apparently healthy animals, 21 were from animals with known intestinal disease at the time of sample collection, and 4 were from animals that developed CE within 1 year of sampling, yielding a total of 25 isolates from animals that had or developed intestinal disease. Culture-positive results were grouped distinctly by animal room of residence and age group (Fig. 1). Juvenile and peripubertal age groups contained the largest proportion of positive cultures. Prevalence data, age, and sampling characteristics for each room are shown in Table S1. Matrix-assisted, laser desorption and ionization, time-of-flight (MALDI-TOF) species identification of the 111 isolates yielded 103 *C*. *coli* isolates (38%, 103/271) and 8 *C. jejuni* isolates (2.95%, 8/271). Of the samples from animals with intestinal disease at the time of sample collection, 23 isolates were *C. coli* and 2 were *C. jejuni*. Both *C. jejuni* isolates from symptomatic animals were obtained from animals diagnosed with CE. Sixteen of the *C. coli* isolates from symptomatic animals were obtained from animals diagnosed with CE, two were from animals with acute colitis, and one was from an animal with colon carcinoma. Four *C. coli* isolates were from animals diagnosed with CE following sample collection.

A summary of the antimicrobial resistance profiles and associated minimum inhibitory concentration (MIC) data is shown in Table 2. Eighty-three of the isolates (74.7%) were sensitive to all antimicrobials tested and 28 (25.2%) were resistant to at least one antibiotic class. A total of 21.6% of the isolates displayed resistance to quinolones, 7.2% to tetracyclines, 3.6% to macrolides, and 3.6% to lincosamides. There was no disagreement between drugs of the same class. None of the isolates were resistant to amphenicols or aminoglycosides. Isolates that displayed resistance to macrolides or lincosamides were invariably also resistant to quinolones and tetracyclines and were considered multidrug resistant (MDR). MDR strains were obtained only from animals with intestinal disease at the time of sample collection.

**TABLE 2:** Summary of MALDI-TOF species identification, antimicrobial susceptibility phenotypes, and MIC data from the obtained 111 *Campylobacter* sp. isolates^*a*,*b*^

Species (*n*) and characteristic	Antimicrobial class susceptibility									
	Susceptible^*c*^	MDR	Quinolone		Macrolide		Amphenicol	Aminoglycoside	Lincosamide	Tetracycline
			Ciprofloxacin	Nalidixic acid	Azithromycin	Erythromycin	Florfenicol	Gentamicin	Clindamycin	Tetracycline
% of all isolates	74.70%	3.60%	78.38%	78.38%	96.40%	96.40%	0%	0%	96.40%	92.79%
*C. coli* (103)										
% of isolates	74.80%	3.80%	77.70%	77.70%	96.20%	96.20%	0%	0%	96.20%	93.30%
MIC range tested	NA^*d*^	NA	0.015–64	4–64	0.015–64	0.03–64	0.03–64	0.12–32	0.03–16	0.06–64
Resistance breakpoint	NA	NA	≥1	≥32	≥1	≥32	≥8	≥4	≥2	≥16
MIC range of isolates	NA	NA	0.06 - ˃64	8 - > 64	0.03 - > 64	0.12 - > 64	0.5–2	0.25–1	0.12–16	0.12 - > 64
MIC mode	NA	NA	0.12	≤4	0.06	1	1	0.5	0.25	0.25
MIC50	NA	NA	0.12	≤4	0.06	0.5	1	0.5	0.25	0.25
MIC90	NA	NA	64	64	0.12	1	1	1	0.5	1
*C. jejuni* (8)										
% of isolates	75%	0%	87.50%	87.50%	0%	0%	0%	0%	0%	87.50%
MIC range	NA	NA	0.015–64	4–64	0.015–64	0.03–64	0.03–64	0.12–32	0.03–16	0.06–64
Resistance breakpoint	NA	NA	≥1	≥32	≥0.5	≥32	≥8	≥4	≥1	≥16
MIC range of isolates	NA	NA	0.06–8	≤4 - > 64	≤0.015–0.03	0.12–0.25	0.5–1	0.25–0.5	0.016–0.12	0.06–32
MIC mode	NA	NA	0.06	4	0.03	0.25	0.5	0.5	0.12	0.12
MIC50	NA	NA	0.06	4	0.03	0.25	0.5	0.5	0.12	0.12
MIC90	NA	NA	8	64	0.03	0.25	1	0.5	0.12	32

^*a*^ Percentages are expressed as percent of isolates of the indicated *Campylobacter* species.

^*b*^ All MIC data are reported in μg/mL.

^*c*^ Susceptible to all tested antimicrobials.

^*d*^ Abbreviations: MALDI-TOF, matrix assisted laser desorption ioniziation time of flight; MDR, multidrug resistant; MIC, minimum inhibitory concentration; NA, not applicable.

**Fig 2:**
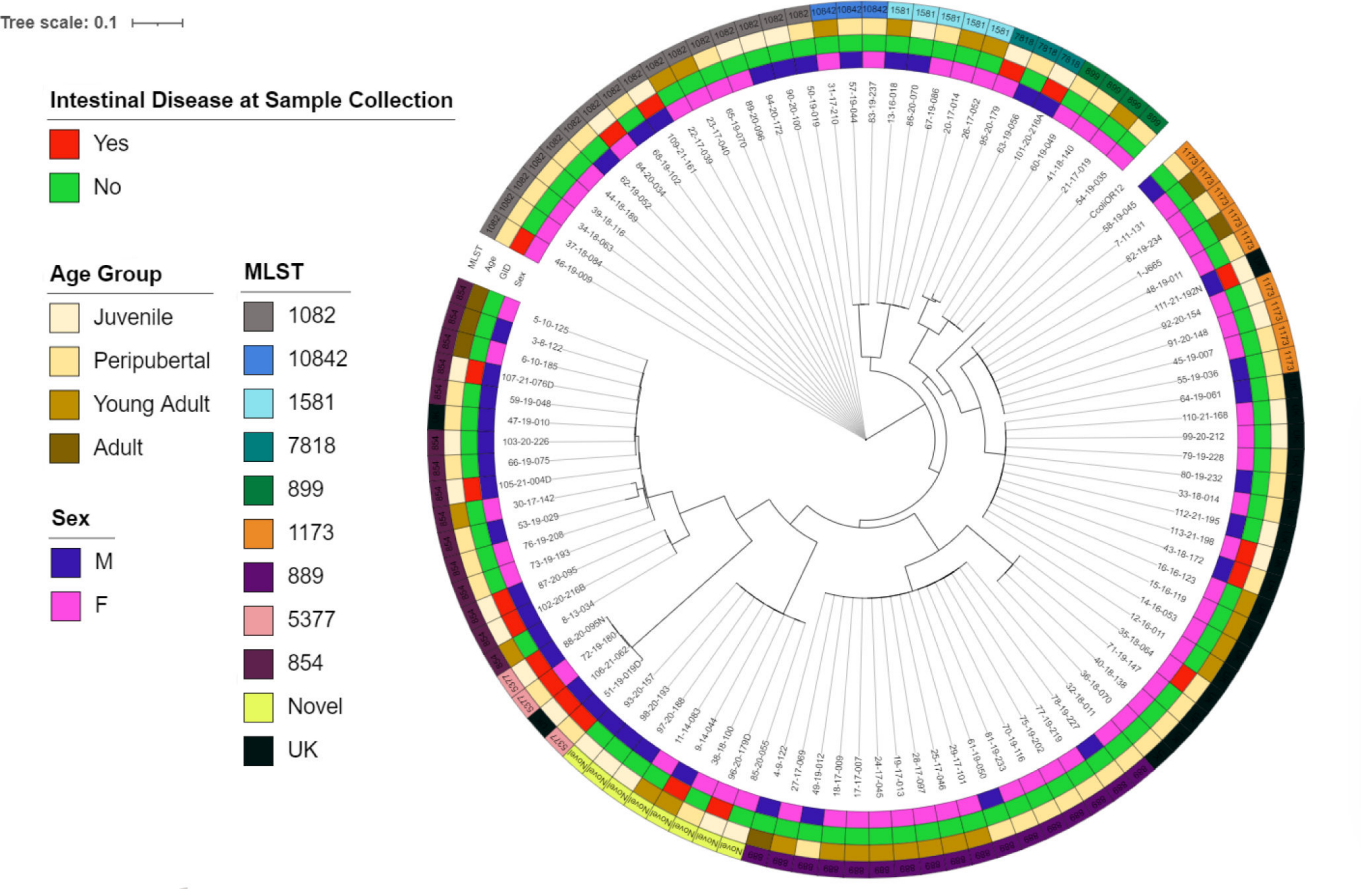
Phylogenetic tree of obtained *C. coli* isolates rooted to the representative *C. coli* OR12 (GenBank accession no. CP013733) strain with annotations for the age group, sex of the animal, clinical status of the animal at the time of sample collection, and MLST of the isolate. UK, unknown MLST as determined by automated methods; GID, intestinal disease at the time of sample collection.

WGS of the isolates yielded *Campylobacter* genome sequences between 1.6 Mbp and 1.8 Mbp. Characterization of the sequences is available in Bacon et al. (31). Examination of phylogenetic relationships (Fig. 2) revealed a non-clonal population with some clustering by animal room (data not shown), age, sex, and clinical status of the animal. Two novel multi-locus sequence types (MLST) were identified (Table S2). Seven sequence types (STs) across 22 isolates were not identified by automated methods; however, using phylogenetic analysis, three STs grouped identically with known MLSTs, and the remainder were across two distinct groups. The vast majority of MLSTs were classified as host generalists. One isolate (MLST 10842) has been primarily reported in poultry. The *C. coli* MLST 5377 group contained all the multidrug-resistant isolates and was isolated from animals with intestinal disease. Three of four of these animals had a history of treatment with multiple antibiotics prior to sampling, though one animal had no record of antibiotic treatment. Three of these isolates, including the isolate from the animal with no history of antibiotic treatment, were clonal isolates, with the fourth very closely related as shown in Fig. 2. *C. coli* isolates from MLST groups 889, 899, 1581, and 10842 were only obtained from healthy individuals.

Regarding genotypic antimicrobial resistance profiles (Table S2), 13 isolates were eliminated from this portion of the analysis due to low coverage, though all isolates were assessed for known point mutations conferring resistance. Two genes for beta-lactam antibiotic resistance were detected, with *bla_OXA-193_* detected in both *C. jejuni* and *C. coli* and *bla_OXA-489_* detected only in *C. coli*. Two genes for aminoglycoside resistance were detected, with *aadE-Cc* found in *C. coli* only and *aph(3′)-III* found in both species. The *tet(O*) gene for tetracycline resistance was detected in both species. The *23s r.2075A>G* point mutation which confers macrolide resistance was detected only in *C. coli*, the *gyrA_2 p.T861* point mutation which confers quinolone resistance was detected in both species, and the *gyrA_2 p.C861* which also confers quinolone resistance was only detected in *C. jejuni*. Agreement between phenotypic resistance profiles and genotypic predictions of resistance was 100% for ciprofloxacin resistance, 99.1% for erythromycin with one isolate phenotypically resistant but lacking the associated point mutation, and 98.0% for tetracycline resistance with two isolates displaying phenotypic resistance but lacking the *tet(O*) gene. The greatest disagreement was with regards to gentamicin with only 79.6% match between phenotypic and genotypic resistance profiles. All isolates were phenotypically susceptible to gentamicin, but twenty isolates contained one of two identified resistance genes. The minimum, maximum, and most frequently reported MIC were identical between isolates that carried aminoglycoside resistance genes and those that did not.

Ninety-seven virulence genes were identified across the 111 isolates. However, the only virulence gene known to play a role in PI-IBS development is *Cdt* (16, 17, 32, 33). All eight *C. jejuni* isolates had all three (A, B, and C) subunits of *Cdt*. The full virulence factor data set for the *C. jejuni* isolates is available in Table S3. Virulence factor assessment of *C. coli* isolates was hampered as the virulence factor database (VFDB) uses *C. jejuni* as the reference genome. Manual, individualized sequence evaluation revealed a 99.6% to 100% sequence match of known *C. coli Cdt* subunit A, B, and C sequences to each of the 103 *C*. *coli* isolates, indicating the presence of the genes for each subunit of Cdt (34).

### qPCR results

**Fig 3:**
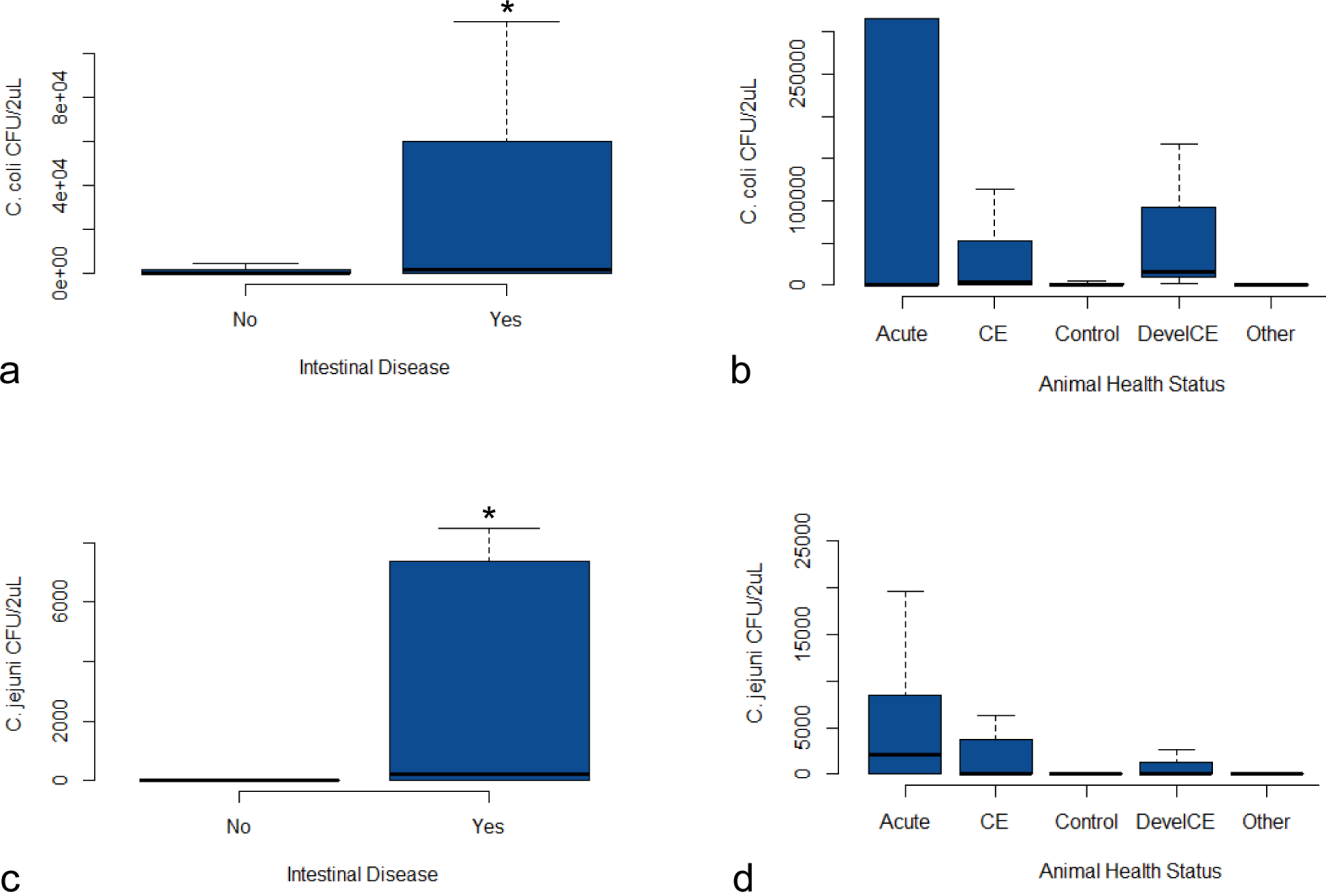
Bacterial quantities, expressed as colony forming units (CFUs) per 2µL of DNA extract, compared across categories of host health status. The “Other” category includes animals with colon carcinoma and intestinal amyloidosis. Significant difference (*P* ˂ 0.05) is denoted by an asterisk. (a) *C. coli* quantity distributed by disease status at the time of sample collection, (**b**) *Campylobacter coli* quantity distributed by specific disease category (significance not assessed due to small categorical sample sizes), (**c**) *Campylobacter jejuni* quantity distributed by disease status at the time of sample collection, and (**d**) *C. jejuni* quantity distributed by specific disease category (significance not assessed due to small categorical sample sizes).

Two hundred and forty-one samples were tested with qPCR. All tested samples had adequate extraction control (Xeno Internal Control) and sampling control (*OSM*) Ct values and were included for further analysis. Four samples had discordant results over two rounds of qPCR for either *C. jejuni* or *C. coli* and were removed from the referable portion of the analysis. Only the first swab taken from each animal was included, resulting in 236 samples analyzed. Similar to the culture results, animals positive for either *C. coli* or *C. jejuni* using qPCR were grouped by room and by age, though with slightly more mixed results using qPCR. Again, the majority of positive results were obtained from juvenile and peripubertal animals (Fig. 1; Table S1). The *C. coli* qPCR prevalence was 45.9%, and the *C. jejuni* qPCR prevalence was 29.6%. 24.9% of samples were qPCR positive for both species. The quantity of bacteria detected was significantly higher (*P* < 0.05) for both *C. coli* and *C. jejuni* in animals with intestinal disease at the time of sample collection compared to animals that were healthy at collection, including those that developed CE following sampling (Fig. 3). The number of individuals in separate disease categories was too low to determine significant differences between intestinal disease groups. However, in addition to increases in animals with acute colitis or CE, animals that developed CE after sample collection also had increased bacterial loads compared to healthy animals (Fig. 3).

### Risk factor analysis

Using logistic regression and controlling for room as a random effect, variables were tested to determine potential risk factors for the presence of *Campylobacter* sp. using either culture or qPCR, and to determine if *Campylobacter* sp. presence alone was a risk factor for the presence of intestinal disease (Table 3). The only risk factor associated with *Campylobacter* presence was age, with juvenile and peripubertal animals at increased risk for the presence of *Campylobacter* sp. using either culture or qPCR. Odds ratio (OR) for juvenile animals using culture was 19.88 (95% CI = 3.59–110.19), OR for peripubertal animals using culture was 47.04 (95% CI = 7.54–293.33), OR for juvenile animals using qPCR was 88.48 (95% CI = 10.66–734.33), and OR for peripubertal animals using qPCR was 33.89 (95% CI 7.25–158.47). Similarly, the only risk factor associated with intestinal disease was also age, with juveniles at increased risk (OR = 55.88, 95% CI = 2.93–1,067.12).

**TABLE 3:** Results of bivariable and logistic regression analysis of potential risk factors for *Campylobacter* sp. presence via culture or PCR and potential risk factors for the presence of gastrointestinal disease at the time of sample collection^*a*^^,^^*b*^

Risk factor	Bivariable analysis, *P* value	Number tested	Number positive (%)	Logistic regression analysis			
				Odds ratio	LL 95%CI	UL 95%CI	*P* value
Culture positive							
Room	6.56E−16						RE
Sex	0.046						
Female		189	69 (36.5)				Referent
Male		77	30 (50.6)				0.109
Age	2.20E−16						
Juvenile		43	26 (60.5)	19.88	3.59	110.19	0.001
Peripubertal		63	51 (80.9)	47.04	7.54	293.33	3.73E−05
Young adult		83	24 (28.9)				0.195
Adult		77	7 (9.09)				Referent
GI disease at collection	0.332	48	16 (33.3)				NI
PCR positive *Campylobacter* sp.							
Room	1.91E−12						RE
Sex	0.272						
Female		168	79 (47.0)				NI
Male		66	37 (56.0)				NI
Age	2.20E−16						
Juvenile		32	29 (90.6)	88.48	10.66	734.33	3.30E−05
Peripubertal		58	49 (84.5)	33.89	7.25	158.47	7.57E−06
Young adult		80	28 (35.0)				0.136
Adult		64	10 (15.6)				Referent
GI disease at collection	0.009	20	16 (80.0)				0.239
PCR positive *C. coli*							
Room	1.08E−11						RE
Sex	0.352						
Female		168	73 (43.5)				NI
Male		66	24 (51.5)				NI
Age	2.20E−16						
Juvenile		31	28 (87.5)	76.69	9.34	630.08	5.37E−05
Peripubertal		58	44 (75.9)	21.69	4.59	102.62	1.04E−04
Young adult		80	25 (31.3)				0.309
Adult		64	10 (15.6)				Referent
GI disease at collection	0.013	20	15 (75.0)				0.324
GI disease at time of collection							
Room	2.20E−16						RE
Sex	0.02						
Female		189	27 (14.3)				Referent
Male		77	21 (27.3)	4.21	1	17.74	0.05
Age	3.73E−09						
Juvenile		43	21 (48.8)	55.88	2.93	1,067.12	0.008
Peripubertal		63	7 (11.1)				0.455
Young adult		83	3 (3.6)				1
Adult		77	17 (22.1)				Referent
Culture positive	0.332	108	16 (13.8)				NI
PCR positive either	0.009	116	16 (13.8)				0.583
PCR positive both	0.008	55	10 (18.2)				0.585
PCR positive *C. jejuni*	0.01	64	11 (17.2)				0.867
PCR positive *C. coli*	0.013	107	15 (14.0)				0.807
History of antimicrobial use	0.147	161	34 (21.1)				0.052

^*a*^ LL, lower limit; UL, upper limit; GI, gastrointestinal; NI, not included in logistic regression analysis; RE, random effect.

^*b*^ 2.2E−16 indicates <2.2E−16.

### Comparison of culture and qPCR methods

Prevalence calculated using qPCR was higher than prevalence with culture across all categories, with the prevalence of *C. jejuni* calculated using qPCR significantly higher (*P* < 0.05) than that calculated using culture. Prevalence of *C. coli* was 38.0% using culture and 46.3% with qPCR, and prevalence of *C. jejuni* was only 2.5% using culture but was 29.6% with qPCR. Using the kappa statistic to compare the two tests, qPCR was in moderate agreement with culture results with respect to *C. coli* (ĸ = 0.67) and there was none to slight agreement between the two tests regarding *C. jejuni* (ĸ = 0.12).

## DISCUSSION

We characterized the *Campylobacter* spp. circulating in a closed specific pathogen free (SPF) breeding colony of rhesus macaques in the United States using culture, WGS, and qPCR. We used these results to determine if any species or strain-specific factors could be identified as associated with acute colitis or CE. Previous non-human primate (NHP) colony surveys for *Campylobacter* have been limited to species level detection, limited antimicrobial susceptibility profiles, and evaluation of the presence of Cdt (5, 8, 26). WGS studies have previously been limited to single genome reports, a selection of isolate sequences without specific regard to health status, or as part of a larger phylogenetic study (5, 27, 35, 36). A rhesus macaque colony survey from Brazil in 2007 found infants had higher susceptibility to the bacteria than adults, males tended to be infected more than females, and *C. jejuni* and *C. coli* displayed sensitivity to nalidixic acid but resistance to cephalothin (8). A similar study in cynomolgus macaques (*Macaca fascicularis*) demonstrated variable resistance profiles in both *C. jejuni* and *C. coli* including resistance to erythromycin, tetracycline, ciprofloxacin, and amoxicillin (37). While these general characterizations are important, they do not specifically assess *Campylobacter* spp. with regard to the health status of the infected individual.

Of the samples tested using culture in this study, 41.8% grew either *C. coli* or *C. jejuni* with no other species isolated. Both species were obtained from healthy animals and from those with intestinal disease. Standard clinical *Campylobacter* sp. isolation techniques likely select for *C. coli* and *C. jejuni* as thermophilic species and may miss non-thermophilic species, many of which are involved in human disease processes (2). Due to laboratory procedures for colony selection, only one isolate per sample was obtained, though a previous study using multiple characterization methods showed pig-tailed macaques (*Macaca nemestrina*) can experience mixed infections, which we also observed in our rhesus macaques using qPCR (6). Regarding antimicrobial susceptibility profiles, 21.6% of isolates were resistant to quinolones and 3.6% were resistant to macrolides, similar to resistance patterns of concern in isolates from the United States human population (38–40). MDR isolates were only isolated from animals with intestinal disease. Interestingly, these MDR isolates were contained within the ST 5377 group, which has also been reported as a consistently MDR ST in antibiotic-free swine production systems (41). Three of the four animals with MDR isolates were treated with macrolide antibiotics prior to sampling; however, only one was treated prior with a quinolone antibiotic, none were treated with tetracyclines or lincosamides, and one showed no record of antibiotic treatment. These results suggest empiric use of quinolones and macrolides in rhesus macaque colonies should be limited to preserve their utility. Antibiotic therapy in animals with intestinal disease should be directed based on isolate-specific antimicrobial susceptibility testing. Additionally, quinolones and macrolides may be less likely to be useful in suspected cases of acute campylobacteriosis.

Using WGS, the isolates were further characterized with regard to their MLST, phylogenetic relationships, genotypic antimicrobial susceptibility patterns, and certain virulence factors. Thirteen MLSTs, primarily host-generalists, were identified, indicating non-clonal strains, a somewhat unexpected finding given the closed nature of the colony. Some MLSTs were found only in animals with intestinal disease, and some MLSTs were only obtained from healthy animals, though the number of isolates from each MLST was considered too small to determine if this was a statistically significant predictive pattern. This heterogeneity does suggest that determining if certain MLSTs are predictive of disease status is a valuable future avenue of investigation. Historically, epidemiologic investigations in humans have shown no particular associations between *Campylobacter* MLST and overall virulence, but recent investigations showed certain MLSTs may predispose to PI-IBS; this should be explored with rhesus macaques and CE (19, 42).

The genetic profiles for antimicrobial resistance tended to match the phenotypic resistance profiles for the isolates, except in the case of genes for aminoglycoside resistance. No isolates demonstrated phenotypic resistance to aminoglycosides, but an entire phylogenetic cluster did contain genes which would predict aminoglycoside resistance, either *aadE-Cc* or *aph(3′)-III*. Both genes independently are known to confer resistance in *Campylobacter* spp., though they may occur within the same isolate, and other studies have noted a similar mismatch (43, 44). This suggests that the genes are either not expressed or are dysfunctional/non-functional, and future studies may investigate the cause of this mismatch (45). A small number of isolates also displayed phenotypic resistance for erythromycin or tetracycline but without the associated point mutation or gene, likely explained by other known resistance mechanisms in *Campylobacter* sp., including the CmeABC efflux pump and altered membrane permeability (43, 46, 47).

Regarding virulence factors, previous studies suggest CdtB as important in the development of PI-IBS (16–18, 48). All isolates contained genes for all three subunits of *Cdt*, consistent with both *C. jejuni* and *C. coli* being pathogens, though *in vitro* studies are required to test the functionality of these genes. While *C. jejuni* and *C. coli* are genetically similar, work focusing on evaluating other potential virulence factor differences in *C. coli* strains from healthy animals versus animals with intestinal disease was hampered by the VFDB utilizing *C. jejuni* as the reference genome. As demonstrated by the investigations into *Cdt*, sequences can vary enough between the two organisms to suggest interpreting virulence factor results for *C. coli* with caution. Future work will investigate these potential differences individually with particular attention to genes that are known to play a role in the development of PI-IBS (19). Except for the recent work investigating *C. jejuni* genotypes associated with PI-IBS, whole genome sequencing of *Campylobacter* spp. in humans and other veterinary species has been primarily used in outbreak related epidemiologic investigations. While we did not obtain enough isolates from animals that went on to develop CE after sample collection to determine specific strain differences that may result in CE, we did demonstrate strain differences between animals with intestinal disease and healthy animals and identified future avenues of investigation which may allow the identification of novel biomarkers or therapeutic targets in rhesus macaque acute colitis and CE.

We also validated qPCR assays for the detection of *C. jejuni* and *C. coli* in rectal swabs from rhesus macaques. Using qPCR, we identified a higher prevalence of animals infected with either species when compared to culture, and co-infection with *C. coli* and *C. jejuni* was identified in 24.9% of samples. Most surprisingly, 29.6% of samples were positive for *C. jejuni* using qPCR, while only 2.5% of samples were positive for *C. jejuni* on culture. Given the large portion of coinfections on qPCR, this is likely driven by colony selection bias during the culture process in a clinical setting. In the authors’ experience, *C. coli* colonies tend to grow more robustly than *C. jejuni* colonies at 42°C, though with the same general colony morphology, and so would be more likely to be selected for speciation and subculturing. Ct values for *C. coli* tended to be higher than those for *C. jejuni* which would support this observation. This effect could be mediated by speciating and subculturing multiple potential *Campylobacter* colonies from the same plate over a more prolonged period, but this would significantly increase the burden on clinical diagnostic laboratories and may not be feasible in all settings. While culture and isolation are required for antimicrobial susceptibility testing and robust genomic analyses, we propose the use of qPCR as a rapid, reliable diagnostic method for campylobacteriosis in rhesus macaques. Ideally, culture and molecular methods will be used simultaneously as the information provided is complimentary. qPCR also allows bacterial quantification. We found significant increases in *Campylobacter* quantity in animals with intestinal disease versus healthy animals, which appears to be driven largely by the vast quantities of bacteria in some animals with acute colitis, and more moderate increases in animals with CE. The increase of *Campylobacter* quantities in animals with CE is likely consistent with the dysbiotic state associated with this disease process. Additionally, while sample sizes did not allow statistical evaluation, a trend of increased bacterial quantity in animals that were healthy at the time of sample collection and went on to develop CE suggests that further study could yield bacterial quantity as a potential diagnostic or even prognostic factor for CE.

The final component of this study was to determine if there were identifiable risk factors for bacteria presence or for the presence of intestinal disease at the time of sample collection. While the studied colony is primarily used for breeding and sale purposes, resulting in an overall colony distribution skewed toward older females, attempts were made to distribute sampling evenly across age groups and both sexes. The male-to-female sampling ratio was 0.4. Using generalized linear mixed model (GLMM), the only significant risk factor for the presence of *Campylobacter* was age, with juveniles and peripubertal animals at increased risk, and similarly, the only significant risk factor for the presence of intestinal disease at the time of sample collection was age, with juveniles at increased risk. While the increased risk was significant for these groups compared with that of adults, the CI of the odds ratios was very wide, limiting our ability to precisely quantify the magnitude of the risk. Younger age as a risk factor for campylobacteriosis in humans has been primarily related to increased environmental exposure and less robust innate immunity; however, successful vaccine studies in NHPs indicate that some degree of accumulation of acquired immunity to individual strains could play a role as well (5, 6, 28, 49–51). As the majority of the isolates in this study were *C. coli*, these results, in conjunction with the bacterial quantity findings, may support *C. coli* as a more opportunistic pathogen, though the obtained *C. coli* isolates did contain genes for toxin production. Similarly, co-infection of *C. jejuni* and *C. coli* was not identified as an independent risk factor for the presence of intestinal disease, though following these co-infected animals to determine if they are more likely to present later with CE will be interesting. As most of the animals with intestinal disease in this study were already diagnosed with CE, a future prospective study focusing more specifically on animals with acute colitis will be valuable. These results do suggest that animals less than 5 years old would be the most valuable targets for future studies on *Campylobacter* sp. in rhesus macaques and potential *Campylobacter* sp. mitigation efforts.

In summary, these findings provide a comprehensive characterization of *Campylobacter* sp. circulating in a breeding colony of rhesus macaques in the United States, including WGS data of 111 isolates, and support the utilization of qPCR for the detection of *C. coli* and *C. jejuni* in conjunction with culture for diagnosis of campylobacteriosis. Future studies will focus on using these tools in animals with acute colitis to identify if any *Campylobacter-*specific factors may play a role in the development of CE following an episode of acute campylobacteriosis, as seen with PI-IBS in humans.

## MATERIALS AND METHODS

### Animals and sample collection

The Keeling Center for Comparative Medicine and Research (KCCMR) at The University of Texas MD Anderson Cancer Center is an Association for Assessment and Accreditation of Laboratory Animal Care (AAALAC) accredited facility where animals are cared for in accordance with the USDA Animal Welfare Act, the *Guide for the Care and Use of Laboratory Animals*, and established Institutional Animal Care and Use Committee policies (52). Research was conducted under the oversight of an Institutional Animal Care and Use Committee (IACUC), protocols #01437-RN03 and #0804-RN03. Animals were part of a closed Indian-origin rhesus macaque (*Macaca mulatta*) breeding colony (*n*, ~1,000) SPF for Simian immunodeficiency virus, Simian retrovirus type I, Simian T-lymphotropic virus, and herpes B virus since 1991. Animals were housed in covered outdoor gang-cages or “corn crib” structures each housing a single small breeding group or a larger group of juvenile and peripubertal animals, each with a designated room number. Paired rectal swabs were obtained opportunistically from 266 animals during sedated (10 mg/kg ketamine) annual health evaluations, while hospitalized for diarrhea, or during necropsy evaluation. One swab was placed in a Cary-Blair Agar BD BBL CultureSwab Transport System (Fisher Scientific, Waltham, MA, USA) or Amies Remel BactiSwab Gel Collection and Transport Swab (Fisher Scientific, Waltham, MA, USA) for culture and the other was placed in an empty sterile microcentrifuge tube for DNA extraction and qPCR. Occasionally, samples were obtained from an individual at multiple time points or only a single swab was obtained from a single time point. If only a single swab was obtained, it was directed for either culture or qPCR. Attempts were made to distribute sampling evenly across all age groups and both sexes, but opportunistic sampling did not allow for true systematic or random selection of subjects. Animals’ health status was assigned upon review of medical records following data collection, and age groups were defined as follows: juvenile, ≤2 years old; peripubertal, 3–4 years old; young adult, 5–9 years old; and adult, ≥10 years old.

### Culture methods

Rectal swabs directed for culture were processed on the day of collection. Swabs were used to inoculate Campy Blood Agar Blaser with 5% Sheep Blood and Antibiotics plates (Fisher Scientific, Waltham, MA, USA) and incubated at 41°C–43°C in a microaerophilic environment for up to 72 hours. Plates with characteristic growth were screened for *Campylobacter* spp. using a Gram stain and oxidase test. Positive isolates were sub-cultured into *Brucella* broth (Becton, Dickinson and Company, Franklin Lakes, NJ, USA) with 10% glycerol added and frozen at −80°C prior to transport from KCCMR to the Texas A&M Clinical Microbiology Laboratory. Isolates were revived on either BD BBL Trypticase Soy Agar with 5% Sheep Blood (Becton, Dickinson and Company, Franklin Lakes, NJ, USA) or Blood Free *Campylobacter* Selectivity Agar (Himedia Laboratories Private Limited, Maharashtra, India) plates incubated at 42°C for 48 hours in a microaerophilic environment. Isolates were speciated using MALDI-TOF mass spectrometry (Biotyper, Bruker, Billerica, MA, USA). Antimicrobial susceptibility testing was performed using a commercial drug panel (Sensititre CAMPY AST Plate; Thermo Scientific, Cleveland, OH, USA) which tested for sensitivity to azithromycin, erythromycin, ciprofloxacin, nalidixic acid, clindamycin, florfenicol, gentamicin, and tetracycline according to the manufacturer’s recommendations (53). CLSI breakpoints for erythromycin and tetracycline were used (54). Categories of susceptible or resistant for the remaining tested antibiotics were determined using the National Antimicrobial Resistance Monitoring System for Enteric Bacteria guidelines (55).

For WGS, cultured isolates were processed and analyzed using the commercial QIAcube HT DNA extraction platform (Qiagen, Germantown, MD, USA), DNA Technologies XGEN Normalase DNA Library Prep Kit EZ and xGen UDI primers (Integrated DNA Technologies, Coralville, IA, USA), the Illumina MiSeq platform, and an established bioinformatics pipeline with the High-Performance Research Computing system at Texas A&M University, as described previously (31). Isolate sequences are available in NCBI under the BioProject accession number PRJNA1054170. Maximum likelihood phylogenies for the *C. coli* isolates were created by running assemblies through Parsnp v.1.2 and FastTree2 using the complete reference genome of a *C. coli* strain OR12 isolate from NCBI (GenBank accession no. CP013733) (56, 57). Individual *C. coli* isolate sequences were compared against known sequences for each subunit using the BV-BRC BLAST function, specifically to evaluate the presence or absence of genes for the three subunits of Cdt in *C. coli* (34).

### DNA extraction of rectal swabs for qPCR

Rectal swabs intended for qPCR were stored at −80°C until the time of DNA extraction, with a collection to extraction interval of 1–7 months. Samples were thawed at room temperature, and DNA was extracted from each swab using a commercial kit (QIAamp Power Fecal Pro DNA Kit; Qiagen, Germantown, MD, USA). The initial CD1 reagent was added to the microcentrifuge tube containing the swab and vortexed for 1–2 minutes to loosen and homogenize the fecal material from the swab. The remaining fluid was pressed from the swab, and the swab was removed. The resulting solution was added to the provided PowerBead Pro tubes, and 4 µL VetMax Xeno Internal Positive Control (Thermo Fisher Scientific, Cleveland, OH, USA) was added to each sample as an extraction efficiency control. The remainder of the procedure was followed per the manufacturer’s instructions. An empty tube was included in each round of extractions as a negative extraction control. Eluted DNA was stored at −80°C and shipped to the Texas A&M University Clinical Microbiology laboratory where it was stored at −20°C until qPCR.

### qPCR

We validated two multiplex qPCR assays for DNA extracted from rhesus macaque rectal swabs, one for the *C. jejuni gyrA* gene and one for the *C. coli cadF* gene. All qPCR was performed using the Applied Biosystems 7500 Fast Real-Time PCR System with associated 7500 Fast SDS v2.3 software (Thermo Fisher Scientific, Cleveland, OH, USA). Primers and probes were obtained from Sigma-Aldrich (St. Louis, MO, USA) and are listed in Table 4.

**TABLE 4:** Quantitative PCR reagent sequences

Gene target	Reagent	Sequence 5′−3′	Origin
*C. coli cadF*	Forward primer	GAGAAATTTTATTTTTATGGTTTAGCTGGT	(58)
	Reverse primer	ACCTGCTCCATAATGGCCAA	
	Probe	6FAM]CCTCCACTTTTATTATCAAAAGCGCCTTTAGAA[BHQ2]	
*C. coli ceuE* ^*a*^	Forward primer	AAGCTCTTATTGTTCTAACCAATTCTAACA	(59)
	Reverse primer	TCATCCACAGCATTGATTCCTAA	
	Probe	[6FAM]TTGGACCTCAATCTCGCTTTGGAATCATT[BHQ2]	
*C. coli glyA* ^*a*^	Forward primer	AAACCAAAGCTTATCGTGTGC	(60)
	Reverse primer	AGTGCAGCAATGTGTGCAATG	
	Probe	[6FAM]CAACTTCATCCGCAAT[BHQ2]	
*C. jejuni gyrA*	Forward primer	AAGATACGGTCGATTTTGTTCCA	(61)
	Reverse primer	CTACAGCTATACCACTTGAACCATTTAATA	
	Probe	[FAM]TGATGGTTCAGAAAGCGAACCTGATGTTTT[BHQ2]	
NHP *OSM*	Forward primer	CCTCGGGCTCAGGAACAAC	(62)
	Reverse primer	GGCCTTCGTGGGCTCAG	
	Probe	[TAM]TACTGCATGGCCCAGCTGCTGGACAA[BHQ2]	

^*a*^ These primer/probe combinations performed poorly compared to the *cadF* assay and were not used in sample testing.

The *C. jejuni* qPCR assay leveraged methods developed for clinical use in the Texas A&M Clinical Microbiology Laboratory and previously published primer-probe sets (61). Two microliters of each DNA sample and blank was combined with 3.85 µL of nuclease-free water (Invitrogen, Waltham, MA, USA), 5 µL of Taqman Fast Virus 1-step Master Mix 4× (Thermo Fisher, Cleveland, OH, USA), 1.25 µL (500 nM) of forward and reverse primers for the *C. jejuni gyrA* gene, 0.4 µL (100 nM) of the probe for the *C. jejuni gyrA* gene, 1 µL of the VetMaxXeno Internal Positive Control Assay (Thermo Fischer Scientific, Cleveland, OH, USA) as an extraction efficiency control, and 2.5 µL (250 µM) forward and reverse primers for the NHP oncostatin M (*OSM*) gene, and 0.25 µL (100 nM) probe for the NHP *OSM* gene to confirm adequate sampling by the rectal swab collection method, for a total reaction volume of 20 µL per sample. Each set of reactions was run with a standard curve of known quantities of *C. jejuni* ATCC 33560 DNA as a positive control and to allow direct quantification of bacterial amounts in each sample. Two microliters of nuclease-free water was used as a no-template negative control, 2 µL of DNA extracted from rhesus macaque whole blood was used as a positive control for the *OSM*, and 2 µL (1,000 copies/µL) Xeno Internal Control DNA was used as a positive control for the Xeno Internal Control Assay. The reaction mixtures were subject to quantification with the following amplification cycle: 2 cycles at 95°C for 20 seconds, followed by 45 cycles of 3 seconds at 95°C and 30 seconds at 60°C. Samples were run in duplicate and those with appropriate amplification curves referable to the *C. jejuni* standard curve were considered positive for *C. jejuni*, and a bacterial quantity was calculated. Samples displaying disagreement were repeated in duplicate once. Samples continuing to display disagreement were labeled as “suspect” and removed from further analysis. The Xeno assay was considered positive if the Ct value was less than 38. *OSM* was considered positive if the Ct value was less than or equal to the *OSM* positive control, with an average control Ct value of 30.7 and an average sample Ct value of 26.4. Samples negative for either the Xeno internal positive control or *OSM* were removed from further analysis.

A similar referable clinical test was not available for the detection of *C. coli*. Initial tests for primer selection and validation were required, as well as validation of multiplexing capability with the Xeno Internal Control and *OSM* assays. A description of the validation efforts for the *C. coli* assay, resulting in the selection of the *cadF C. coli* target, as well as validation of testing archived samples is available in Supplemental Methods and Results, with supporting tables and figures (Tables S4 and S5; Fig. S1). The final reaction method was 2 µL of each DNA sample and blank combined with 4.35 µL of nuclease-free water (Invitrogen, Waltham, MA, USA), 5 µL of Taqman Fast Virus 1-step Master Mix 4× (Thermo Fisher, Cleveland, OH, USA), 1 µL (300 nM) of forward and reverse primers for the *C. coli cadF* gene, 0.4 µL (100 nM) of the probe for the *C. coli cadF* gene, 1 µL of Xeno Assay, and 2.5 µL (1 µM) forward and reverse primers for the NHP *OSM* gene, and 0.25 µL (100 nM) probe for the NHP *OSM* gene, for a total reaction volume of 20 µL per sample. As with the *C. jejuni* reaction, each set of reactions was run with a standard curve of known quantities of *C. coli* (ATCC 49941), 2 µL of nuclease-free water as a no-template negative control, 2 µL of DNA extracted from rhesus macaque whole blood as a positive control for the *OSM*, and 2 µL Xeno Internal Control DNA as a positive control for the Xeno Internal Control. The reaction parameters were identical to the *C. jejuni gyrA* reaction.

### Statistical analysis

All statistical analyses were performed using R software (version 4.2.2) in R studio, using only results from the first sample from each animal in the case of repeated sampling (63). We calculated prevalence and explored risk factors for *Campylobacter* sp. presence as detected by culture or qPCR, relative to disease and host characteristics using logistic regression with GLMMs, controlling for room as a random effect. Each risk factor was tested for potential significance with *χ*^2^ or Fisher’s exact tests. Risk factors with a *P* value ≤0.25 in bivariable analysis were included in the logistic regression analysis. For risk factors included in logistic regression analysis and subsequently significant (*P <* 0.05), odds ratios, and 95% confidence intervals were calculated. Additionally, to determine if bacterial quantity as detected by qPCR was significantly associated with disease, the bacterial quantity as measured by qPCR was tested for normality using the Shapiro–Wilk test and then subjected to Wilcoxon signed-rank testing for significance. Results were considered significant if *P <* 0.05. For comparison of culture (considered the gold standard) and qPCR methods for detecting *Campylobacter* sp. presence, agreement between methods was tested using Cohen’s kappa (*κ*).

## SUPPLEMENTAL MATERIAL

Supplemental material msphere.00560-24-s0001.pdf Validation of the *Campylobacter* real-time PCR methods; Fig. S1; Tables S4 and S5.

Table S1 msphere.00560-24-s0002.xlsx Details of *Campylobacter* species-positive animals by qPCR or culture distributed by room with median age, age range, and number of animals tested of each room.

Table S2 msphere.00560-24-s0003.xlsx MLST and genotypic antimicrobial resistance data in comparison to phenotypic resistance profiles for each evaluated *Campylobacter* isolate.

Table S3 msphere.00560-24-s0004.xlsx Virulence factors identified in 8 *Campylobacter jejuni* isolates from rhesus macaque rectal swabs.

ASM does not own the copyrights to Supplemental Material that may be linked to, or accessed through, an article. The authors have granted ASM a non-exclusive, world-wide license to publish the Supplemental Material files. Please contact the corresponding author directly for reuse.
