# Supplementary material for: Diversity of Campylobacter spp. circulating in a rhesus macaque (Macaca mulatta) breeding colony using culture and molecular methods
Source: mSphere. 2024 Oct 23;9(11):e00560-24. doi: 10.1128/msphere.00560-24 (PMC11580467; doi:10.1128/msphere.00560-24)

Supplemental Methods and Results

Validation of C. coli qPCR

A referable clinical test was not available for the detection of *Campylobacter coli* using quantitative PCR (qPCR) from rectal swabs, as was available for the detection of *C. jejuni*. Initial tests for primer selection and validation of standard curves were required, as well as the validation of multiplexing capability with the Xeno™ Internal Control and *OSM* assays. Primer and probe combinations selected for trial were *cadF*, *ceuE*, and *glyA*, based on previous publications.[1,3,4,5] DNA extracted from a pure colony of *C. coli* (ATCC 49941) was used to determine the ideal concentration of each primer-probe combination. *CadF* and *ceuE* combinations behaved similarly regardless of the concentration and 300 nM of each primer and 100 nM of each probe were selected as the concentrations for all future work. *GlyA* produced poor amplification regardless of concentration (data not shown) and was removed from further analysis. To select between *cadF* and *ceuE*, each primer-probe set was tested against a standard curve multiplexed with the Xeno™ Internal Control assay. The Xeno™ Internal Control assay performed better in conjunction with the *cadF* primers and probe (Table S4), so *cadF* was selected as the detection method for *C. coli* in the rhesus macaque fecal DNA samples. The *cadF*+ Xeno™ Internal Control assay was also tested for inclusivity and exclusivity against other common *Campylobacter* species. The assay detected four different strains of *C. coli* (BAA370, BAA371, BAA1061, ATCC 49941), and displayed no amplification of DNA from two strains of *C. fetus venerealis* (ATCC 19438, ATCC 33561), two strains of *C. fetus fetus* (ATCC 27374, ATCC 25936), or a strain of *C. jejuni* (ATCC 33560). The *cadF* assay was also multiplexed with the NHP *OSM* assay as above.[2] Each assay was tested at varying ratios of *Campylobacter* to rhesus macaque DNA and the functionality of the three primers (*cadF/gyrA, OSM,* and Xeno™ Internal Control) tested in triplicate (Table S5). Ultimately, these efforts resulted in a final reaction of 2 µL of each DNA sample and blank combined with 4.35 µL of nuclease-free water (Invitrogen, Waltham, MA), 5 µL of Taqman Fast Virus 1-step Master Mix 4x (Thermo Fisher,

Cleveland, OH), 1 μL (300 nM) of forward and reverse primers for the *C. coli cadF* gene, 0.4 μL (100 nM) of the probe for the *C. coli cadF* gene, 1 μL of Xeno™ Assay, and 2.5 μL (1 μM) forward and reverse primers for the NHP *OSM* gene, and 0.25 μL (100 nM) probe for the NHP *OSM* gene, for a total reaction volume of 20 μL per sample. As with the *C. jejuni* reaction, each set of reactions was run with a standard curve of known quantities of *C. coli* (ATCC 49941), 2 μL of nuclease-free water as a no-template negative control, 2 μL of DNA extracted from rhesus macaque whole blood as a positive control for the *OSM*, and 2 μL Xeno™ Internal Control DNA as a positive control for the Xeno™ Internal Control. The reaction parameters were identical to the *C. jejuni gyrA* reaction.

Validation of C. coli and C. jejuni qPCR on archived samples

To assess the utility of the assays with respect to archived samples, validation trials to mimic DNA extraction and qPCR from frozen swabs were performed using canine feces negative for *Campylobacter* species and then spiked with known quantities of *C. coli*. Swabs were spiked and DNA immediately extracted on Day 0. A second set of swabs were spiked at stored for 1 month at -80°C, and a third set was spiked and stored in Amies Remel™ BactiSwab™ Gel Collection and Transport Swab (Fisher Scientific, Waltham, MA) for 1 month at -80°C. DNA extraction was performed on the second and third set on the same day after 30 days of freezing. DNA extract from all three sets of swabs were stored at -20°C and qPCR performed on the same day. The assay behaved similarly at both time points, however there was more variable detection limits in the freezer stored samples, particularly at *C. coli* concentrations ≤ 26,000 CFU/mL (Figure S1). Similar methods were applied to the *C. jejuni* assay (data not shown). Given these results and logistic limitations to performing same day DNA extractions on freshly collected swabs from the rhesus macaques, a higher cycle number (45) was selected to avoid classifying an animal shedding ≤ 26,000 CFU/mL as negative,

limiting potential misclassification of animals which were positive as negative to those shedding ≤ 2,600 CFU/mL.

Supplemental References

1. Best EL, Powell EJ, Swift C, Grant KA, Frost JA. Applicability of a rapid duplex real-time PCR assay for speciation of Campylobacter jejuni and Campylobacter coli directly from culture plates. *FEMS Microbiology Letters*. 2003;229**:** 237-241.

2. Bruce AG, Bakke AM, Thouless ME, Rose TM. Development of a real-time QPCR assay for the detection of RV2 lineage-specific rhadinoviruses in macaques and baboons. *Virology journal*. 2005;2**:** 1-12.

3. Leblanc-Maridor M, Beaudeau F, Seegers H, Denis M, Belloc C. Rapid identification and quantification of Campylobacter coli and Campylobacter jejuni by real-time PCR in pure cultures and in complex samples. *BMC microbiology*. 2011;11**:** 1-16.

4. Tissier A, Denis M, Hartemann P, Gassilloud B. Development of a rapid and sensitive method combining a cellulose ester microfilter and a real-time qPCR assay to detect Campylobacter jejuni and Campylobacter coli in 20 liters of drinking water or low turbidity waters. *Applied and Environmental Microbiology*. 2011.

5. Toplak N, Kovač M, Piskernik S, Možina SS, Jeršek B. Detection and quantification of Campylobacter jejuni and Campylobacter coli using real-time multiplex PCR. *Journal of applied microbiology*. 2012;112**:** 752-764.

Supplemental Tables and Figures

Table S4: Average number of amplification cycles required to cross threshold for each primer set tested in duplicate.

CFU/mL	*ceuE* qPCR		*cadF* qPCR	
	ceuE Ct	Xeno[a] Ct	*cadF* *Ct*	Xeno[a] Ct
18,000,000	20.68	40.00	21.57	33.98
1,800,000	27.10	40.00	27.72	32.98
180,000	31.62	39.59	32.19	34.11
18,000	33.24	35.70	34.52	32.32
1,800	36.52	35.74	38.92	32.77
180	39.89	36.90	40.00	33.90

[a]Applied Biosystems™ VetMAX™ Xeno™ Internal Positive Control DNA and Assay. The Xeno™ Internal Positive Control DNA is added to samples prior to extraction as an extraction and PCR inhibition control.

Abbreviations: CFU/mL, colony forming units per millileter; qPCR, quantitative PCR; Ct, cycle threshold

Table S5: Average number of amplification cycles required to cross threshold for multiplexed qPCR assays tested in triplicate.

***C. coli cadF+OSM* Assay**					
CFU/mL	NHP DNA (ng/mL)	*cadF* qPCR Ct	*cadF* St Dev	*OSM* qPCR Ct	*OSM* St Dev
25,200	0.158	32.39	0.13	31.60	0.28
2,520	0.158	35.53	0.18	31.87	0.28
252	0.158	45.00	0.00	31.68	0.12
25,200	1.58	32.13	0.37	28.48	0.03
2,520	1.58	35.33	0.82	28.62	0.09
252	1.58	45.00	0.00	28.78	0.18

25,200	15.8	32.03	0.17	25.55	0.19
2,520	15.8	40.04	4.38	25.28	0.01
252	15.8	45.00	0.00	25.31	0.07
***C. jejuni gyrA+OSM* Assay**					
CFU/mL	NHP DNA (ng/mL)	*gyrA* qPCR Ct	*gyrA* St Dev	*OSM* qPCR Ct	*OSM* St Dev
6,400	0.158	33.86	0.32	31.16	0.21
640	0.158	39.02	5.18	31.04	0.15
64	0.158	45.00	0.00	30.89	0.26
6,400	1.58	34.17	0.73	27.66	0.51
640	1.58	39.20	5.14	28.17	0.17
64	1.58	45.00	0.00	28.43	0.10
6,400	15.8	33.64	0.15	24.81	0.05
640	15.8	38.69	5.46	24.88	0.10
64	15.8	45.00	0.00	24.59	0.18
***cadF*/*gyrA*+*OSM*+Xeno™ Multiplex Assays[a]**					
	cadF qPCR Ct	23.82	*gyrA* qPCR Ct	14.67	
	cadF St Dev	1.61	*gyrA* St Dev	0.50	
	OSM qPCR Ct[b]	29.63	*OSM* qPCR Ct[b]	30.07	
	OSM St Dev	0.61	*OSM* St Dev	0.08	
	Xeno™ Ct	30.77	Xeno™ Ct	32.16	
	Xeno™ St Dev	0.36	Xeno™ St Dev	0.09	

[a]Bacterial DNA was obtained from direct boiling of several bacterial colonies following 48 hour incubation at 42°C.

[b]NHP DNA concentration used for complete multiplex trials was 1.58 ng/mL.
Abbreviations: CFU/mL, colony forming units per millileter; NHP, non-human primate; OSM, non-human primate oncostatin M; St Dev, standard deviation

Figure S1: Validation of qPCR on DNA extracted from fresh and frozen canine fecal swabs negative for *Campylobacter* sp. and spiked with a known concentration of *C. coli*. DNA was extracted from the swabs directly after spiking (Day 0), following 30 days of freezing at -80°C in no media (Day 30 without media), and following 30 days of freezing at -80°C in Amies Clear media (Day 30 with media), and qPCR for *C. coli* performed as described.

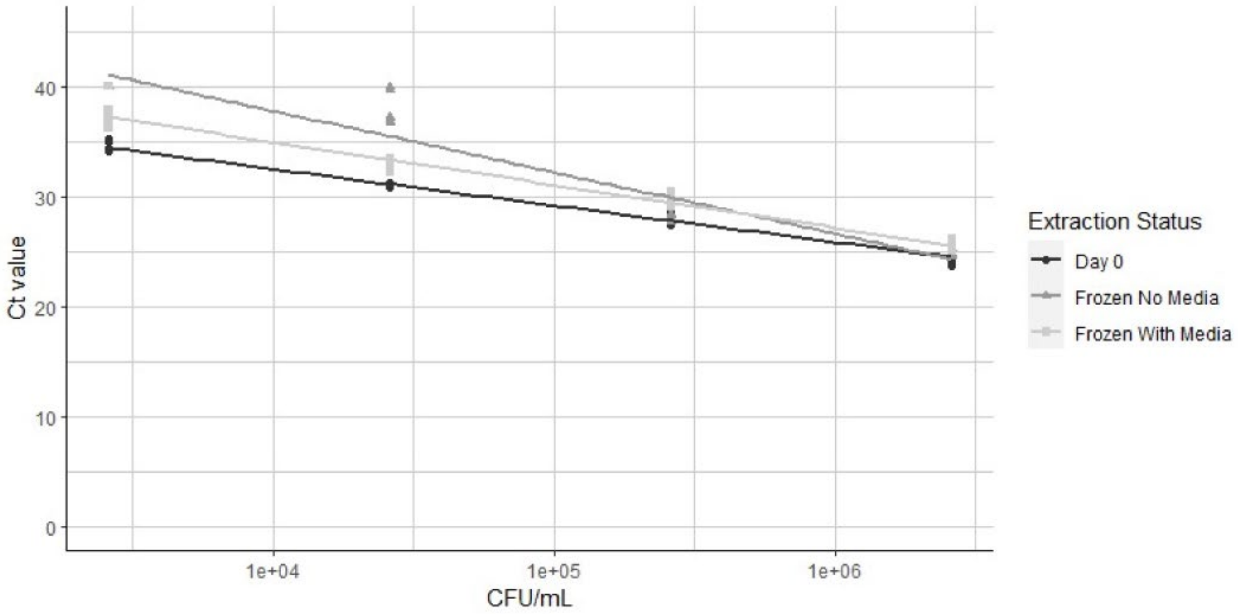

Supplement: Supplemental material — Validation of the Campylobacter real-time PCR methods; Fig. S1; Tables S4 and S5. [file msphere.00560-24-s0001.pdf]
